# Poverty. Its Evils and Their Remedies. Parochial Medical Relief

**Published:** 1840-01-01

**Authors:** 


### POVERTY. ITS EVILS AND THEIR REMEDIES. PAROCHIAL MEDICAL RELIEF.

I. Forty-Fourth, Forty-Fifth, and Forty-Sixth Reports from Select Committee on the Poor Law Amendment Act; with the Minutes of Evidence and Appendixes. (Medical Inquiry.) Ordered by the House of Commons to be printed, 20th and 22d June, 1831. London, fol. pp. 147.

II. Extracts from the Information received by His Majesty's Commissioners as to the Administration of the Poor Law. Published by Authority. London, 1833, 8vo. pp. 432.

III. Report from His Majesty's Commissioners for Inquiring into the Administration and Practical Operation of the Poor Laws. Published by Authority. London, 1834, 8vo. pp. 362.

IV. Four Annual Reports of the Poor Law Commissioners for England and Wales. London, 1835, 6, 7, 8.

V. Plans for Regulating Medico-Parochial Attendance: also, Observations on "Self-Supporting" Dispensaries and Union Infirmaries. By *John Charleton Yeatman.* 4th Edition, enlarged. London, 1837, 12mo. pp. 70.

VI. Medical Relief for the Labouring Classes, on the Principle of Mutual Insurance. By *H. W. Rumsey,* one of the Surgeons of the Chesham Self-supporting Dispensary, and Fellow of the Royal Medical and Chirurgical Society. London, 1837, 12mo. pp. 69.

VII. Mr. Serjeant Talfourd's "Letter" to W. H. Rumsey, Esq. Secretary to the Poor Law Committee of the Provincial Medical and Surgical Association.

The absence of any legislative enactment, providing satisfactory attendance upon the poor in their hour of sickness, and sufficient remuneration for their medical attendants, has hitherto left the case of both to the tender mercies of parochial authorities, whose additional duty of protecting the pockets of the rate-payers in their respective parishes, they have only too generally considered to be the sole public object for which they were appointed to office.

The attempt, under the Poor Law Amendment Act, at indirectly coercing the necessitous poor to provide medical relief for themselves—and the medical profession to afford such relief on the parish account, at the bare cost of the necessary drugs, was an impudent and barefaced mockery of the sick poor, a wicked and unrighteous fraud upon a whole profession.

But the rights of the poor had their advocates, and those advocates were too wary not to perceive the monstrous inhumanity which must accrue from such an attempt, and not to take note of every individual instance of such inhumanity when it occurred. And it could hardly have been expected that an entire profession would remain dormant while their interests were sacrificed throughout the whole country, for no higher purpose than to enable well-salaried Commissioners to get up, from year to year, a fair and prosperous looking balance-sheet; and to send forth, from year to year, voluminous reports of the admirable working of that machinery whereby the sick poor have been neglected or maltreated, and their attendants rewarded in the inverse ratio of their labours. It was too much to be hoped for, even by those Commissioners, that the medical profession should be defrauded, or that the sick poor should be wronged, without remonstrance or murmur; and that while those same Commissioners pocketed their thousands of pounds per annum—the means and appliances of maintaining handsome establishments—and quaffed their Champagne and Burgundy, the poor, as at Bridgewater, should be quietly regaled on barley-water and water-gruel!

But, it is nevertheless true, that not only was such a hope indulged in—it was even thought to have been realized, and, while the fraud upon the medical profession was prospering, and the injury to the sick poor was proceeding without any serious interruption to its consummation—their very material diminution in the land—the Gentlemen of Somerset House were drawing up a congratulatory report, and felicitating themselves on their success.

Appointed by, and subject to, the Commissioners, there is a more numerous body of Assistant-Commissioners; whose business it has been to reduce the respective Boards of Guardians to the moral dimensions, and to confine the amount of medical and other relief awarded the poor in their several Unions, to the pecuniary limitations, prescribed by their own immediate superiors, and to bolster up the worst features of that system which had called themselves into official existence.

The Assistants transmit their district reports of the state of things—the Commissioners avail themselves of their testimony to assure the public that all is well—and Mr. Chadwick prepares an annual report to that effect, garnished with the laudatory garlands wove for his masters' brow, by his masters' deputies.

One of the Assistant-Commissioners, writing from Bucks, observes, "the introduction of any new system, calculated to remove corruption, is attended with many difficulties; and seldom, till after the lapse of some considerable time, can it be got even into operation." "I have, therefore, great reason," he continues, "to congratulate the Board that this measure, the one of all others that had corruption and personal interest to contend against, and where prejudices both of the higher and lower classes united to impede its progress, has not only got into operation, but has so far cleared its way, as in the short space of a few months to produce beneficial results sufficiently powerful to convince the doubtful, convert the hostile, and to unite them in endeavouring to carry its principles more extensively into effect." *First Annual Report of the Poor Law Commissioners, p.* 249.

What as regards medical remuneration, the "principles" were, of "*this measure, which of all others had corruption and personal interest to contend*

*against;*" it would puzzle a Philadelphia lawyer to say, unless, indeed, they were injustice and cruelty.

That the medical profession were *corrupt* enough to wish to take care of their own pence, while the Commissioners were pocketing the country's pounds—and that the poor were sufficiently *self-interested* to shudder at the surrender of their own health and life into the hands of the lowest bidder, was no doubt monstrous and intolerable.

But this part of our narrative is too important to be lightly, or even hurriedly passed over; and we shall transfer from the " dim obscure " of their own report, to the clearer, brighter page of history, for a more open and public perusal, the following morçeau, in elucidation of our patriotic Poor Law Commissioners' " principles "—the principles of that measure, whose well-working, according to the aforesaid veritable witness, W. J. Gilbert, Esq. had convinced the doubtful and converted the hostile, and united them both, to carry it more extensively into effect.

A richer specimen of Loyolan logic, was never coined, or issued from the Vatican.

" § 25. Amongst the evils we found ourselves called upon to remedy, were a large class connected with the administration of medical relief. In our present Report, we deem it necessary only to advert to some of the chief evils of this class, and to the measures we have adopted with relation to them.

We found it a practice, in the great majority of instances, for a medical man to contract with the parish for the supply to the settled paupers of the parish, with medical attendance for a small fixed sum, on the expressed or implied condition that he should be allowed to make whatever charges he pleased for his attendance and treatment of non-parishioners, under suspended orders of removal, or an order of medical relief by the overseer. When the patient has recovered, he is sent home to a parish with a bill for medical attendance, including charges for medicine at the highest rates. Against these charges the distant parish to which the pauper belonged had no adequate protection. The pauper was exposed to the danger of being supplied with medicines considerably beyond what was required for his proper treatment. Instances have come to our knowledge where, in large populous parishes, the profits of attendance upon paupers under such circumstances have been upwards of £300 per annum. The inferior officers have been feed by the medical officer, to search out and give him information of the cases of this description. As a further evil of this system, we may mention that paupers with their families have been removed from their parishes at a great expense, when each head of a family would otherwise have preferred remaining and seeking employment in the parish where his sickness occurred. By countenancing these practices, parishes were in the habit of creating burdens for each other. As a check to this system, and to the general expense of medical relief, we have generally required that medical services should be retained by contract and open tender, including, as a condition, that the medical officers should attend at the same charge all patients on the order of the overseer, whether the patients were parishioners or non-parishioners.

In regulating the appointments of medical officers within the new unions, we have acted on the presumption, that by the words of the Act (that the medical officer shall be ' a person duly licensed to practise as a medical man,') it was intended to include equally physicians, surgeons, or apothecaries, duly licensed to practise as such. Applications have been made to us to prescribe, as the qualification of the medical officer of any Union, that he should be a member of the College of Surgeons as well as of the Apothecaries' Company; but as at present informed, we do not think that the public interest would be advanced by confining the qualification within narrower limits than those traced by the words of the Act, and which conform to the general practice. With respect to the general professional qualifications of the medical men who come within the words of the Act, we have relied on the diplomas of those who are charged by the legislature with the duty of examining the qualifications of the candidates for practice, being assured that the recent improvements in medical practice and education are such as in general to render the later diplomas certificates of a degree of competency, equivalent to much practice on the parts of those who have had an earlier education. Under these circumstances we have considered that the interests of the public and of the profession itself were the best served by keeping the situations of medical officers in the new Unions open to the competition of the whole body of medical practitioners.

Instead of attempting to fix the price of the services of the medical practitioners for the Union, we deemed it the most advantageous that each practitioner should fix the price of his own services, under competition. Amongst the inducements to accept these appointments, are, *the credit* of *the appointment* of medical officer to an Union by a Board of Guardians, the *wider fields* these appointments afford *for the display of care and skill, and for obtaining reputation* leading to more profitable practice; inducements differing in degree, but similar in kind to those upon which men of the most eminent skill find it to their interest to give their services to the chief medical institutions of the country. We may be sure that the medical practitioner will, in fixing upon his terms, do nothing which he considers will not on the whole be advantageous to himself; and next, that he will consider the interests and advantage of his own profession. We have found it necessary, as a security against undue charges even under competition, to adopt as a rule that the aggregate charges for medical relief within the new Unions, shall not exceed the aggregate of the former expenditure for medical relief in the separate parishes now included in the Unions. Instances have occurred where the local medical practitioners have combined to prevent a competition. The course taken in these instances for the protection of the rate payers, and to secure the best treatment to the paupers, has been to suspend our sanction to the appointments, and to cause advertisements to be made to throw open the offices to the competition of practitioners from a distance, or of the profession at large.

In some Unions, as in the Wycombe Union, it has been provided that the terms of the contract should be a remuneration, at a given sum per head, on the number who receive medical relief; but with the proviso, that the gross charge should not exceed a given amount. It is stated to us in evidence, that this mode of proceeding, though adopted reluctantly by the medical profession, has operated very beneficially. The surgeon of the Amersham Union, Mr. Thomas Brickwell states, in the course of an examination,

I approve of the system; but the amount in the present contract is inadequate; I think I shall lose a guinea a week by it. In some of the parishes it is at present only one third of what I have received in former years for the same time. But I approve of the system, for these reasons: it is a self-acting check upon the relieving officer in giving improper orders, or withholding proper orders; upon the applicant for medical relief, in making him feel that in receiving it he is a pauper, and causing the parish a specific charge for him; and upon the medical man, by causing an inquiry into each case, so that none can escape attention; and by that means also secures proper attendance to the patient. Indeed, the mode of contract forms a complete system of check and security in cases of pauper medical relief, the want of which was so much felt under the old system.

Has it tended to curtail the evil of sending all parties to the parish doctor for medical relief, which was so prevalent under the cases of contract in gross?—Yes, it has;—I have many cases now that I am attending as independent patients, who used always, before, to come to me as paupers. One case is that of a woman of Fenn; her son is a master bricklayer, with whom she resides, the cottage and garden their own. She has a daughter about 30, a sempstress, who gets a very good living. This woman was, with her daughter, always attended by the parish. On my telling this woman that the parish paid a specific sum for her, she refused to be attended, and now pays for herself. There are many cases which evidence this effect of the system.

Of course, the new independent patients pay you?—Yes, they do; they pay us at the time they have the medicine.

Now, although you do not receive so much from the parish contract, will not the amount by these new independent patients more than make up the difference? —No, I think not; but it will go towards it.

We anticipate that the introduction of a better system will be beneficial for the destitute sick, as well as beneficial to the labouring classes generally; and that it will be found conducive to all proper interests of the respectable portion of the medical profession.

It will, however, be observed, that the change in the system has not, in many instances, been so long in operation as to develope the whole of the effects which may be anticipated from it, in promoting voluntary and independent associations to provide for the casualties of sickness and mortality.

But even now the reports made to us of the very satisfactory effects of the operation of the rule are becoming daily apparent. We cite the following passage from a recent report made to us by our Assistant-Commissioner, Mr. Hall, as illustrative of the tenor of other incidental communications on the subject:—

The good effects of your arrangements as respects medical relief, are shewing themselves in the shape of medical clubs among the labourers. One of the surgeons of the Wallingford Union told me that several were in process of formation in his district; and I have heard that elsewhere the labouring class has evinced the same degree of foresight and providence; has given the same proof that, when thrown upon his own resources, and taught to rely upon his own exertions, the independent labourer can, and will adopt measures answering to the necessities of the case.

Mr. Gulson states in the recent report from Oxfordshire, 'medical clubs are starting up in all directions. The proceedings of the Board, as regards the medical department, have already been productive of the best results. Highly respectable medical men are undertaking to attend all cases for an annual subscription of 2s. for a single person; and for 4s. 4d. they engage to attend a whole family, however large, so that it does not include children above sixteen years of age. At Witney, Benson, and other places, the labourers are subscribing in considerable numbers to independent medical clubs.' Mr. Gilbert reports to us that in several parts of Buckinghamshire similar effects, resulting directly from the change of medical relief, have been developed in a striking manner."—*First Annual Report of Poor Law Commissioners*, pp. 51 to 55.

Let our readers attentively peruse the first paragraph of the preceding extract, in connexion with the following report from His Majesty's Commissioners for inquiring into the administration and practical operation of the poor, out of which report grew the Poor Law Amendment Act itself, and the Commission under it. The part of that report to which we entreat particular attention is headed:—

"OUT-DOOR RELIEF OF THE IMPOTENT."

"The out-door relief to the impotent (using that word as comprehending all except the able-bodied and their families) is subject to less abuse. The great source of poor-law mal-administration is, the desire of many of those who regulate the distribution of the parochial fund, to extract from it a profit to themselves. The out-door relief to the able-bodied, and all relief that is administered in the workhouse, afford ample opportunities for effecting this purpose; but no use can be made of the aged and sick, and there is little room for jobbing, if their pensions are paid in money. Accordingly we find, that even in places distinguished in general by the most wanton parochial profusion, the allowances to the aged and infirm are moderated.

The out-door relief of the sick is usually effected by a contract with a surgeon, which, however, in general, includes only those who are parishioners. When non-parishioners become chargeable from illness, an order for their removal is obtained, which is suspended until they can perform the journey; in the mean time they are attended by the local surgeon, but at the expence of the parish to which they belong. This has been complained of as a source of great peculation; the surgeon charging a far larger sum than he would have received for attending an independent labourer or a pauper, in the place of his settlement. On the whole, however, medical attendance seems, in general, to be adequately supplied, and economically, if we consider only the price and the amount of attendance." p. 43.

So then, according to a Royal Commission, including within it such men as the present Bishops of London and Chester, and the Right Hon. W. Sturges Bourne, and not even excluding from it the Poor Law Commissioner's Secretary Edwin Chadwick, Esq. medical attendance, under the old system, appears to have been adequately and economically supplied, if the price and the amount of attendance only, be considered. Whereas, the Poor Law Commissioners, eighteen months after, include " amongst the evils they found themselves called upon to remedy," " a large class connected with the administration of medical relief!"

But what are the evils which constitute this " large class." The Commissioners " deem it necessary only to advert to some of the chief" of those " evils;" that is, to *one* and *only one*, as " *some* of the *chief*" evils of this class, and that one is, the complaint already made to the Royal Commission of undue charges on the part of practitioners, for the medical attendance given to non-parishioners! Like the solitary testimony of Mr. Brickwell, one out of thirteen surgeons in the Amersham Union, it is brought ostentatiously forward, to justify an alteration for the worse, when no alteration at all was called for by the public, and none whatever recommended by the Commission of Inquiry.

Then come the " remedies" for " them," that is, in the correct and simple language of truth, for " it." And what a remedy! to reduce medical men to the necessity of putting their skill and humanity up to sale by tender and contract, as the butcher and baker their bread and meat! But, " *we may be sure that the medical practitioner will, in fixing upon his terms, do nothing which he considers will not be on the whole advantageous to himself; and next, that he will consider the interests and advantage of his own profession.*"

Indeed! but it is presently added " instances have occurred where the local medical practitioners have combined to prevent a competition," and what was the " remedy" then? for " we are sure," to borrow the Commissioners' words, they would only do what they considered to be at once advantageous to themselves and their profession. Why, the office was thrown open by advertisement " to the competition of practitioners from a distance, or of the profession at large" and this, of course, altogether " for the protection of the rate-payers," and,—to secure the best treatment to the paupers!" It was not, in the most remote degree, for the purpose of overawing the local medical practitioners, by the prospect of losing part of their private practice, into doing any thing which they were agreed would be disadvantageous to themselves and injurious to the interests of their common profession.

But what were the effects of the " better system," which it was anticipated would be alike " beneficial for the destitute sick" and " to the labouring classes generally," as well as " conducive to all *proper* interests of the *respectable* portion of the medical profession ?" Those excellent results may be seen in the rise and progress of " medical clubs" among the labourers, and the spectacle of " highly respectable medical men, undertaking to attend all cases for an annual subscription of two shillings for a single person ; and four shillings and four pence for a whole family, however large !"

That neither the profession, the poor, nor the public, were either " convinced" or " converted" by the Commissioners' or their Assistants' reports in praise of themselves and their proceedings, subsequent events clearly demonstrated. The parochial practitioner complained that he was hard worked and ill paid. The sick poor cried aloud for more and better help, and complained bitterly that they were either neglected, or treated unskilfully. The public at large, or that part of it which takes an interest in such matters, wondered that their pockets were as often visited, and as fully taxed, as before the introduction of the " better system," and they complained that they did not find their own condition one whit improved by the abridgment of the poor man's physic, or the reduction of the medical officer's salary.

If the following excerpt from a subsequent report does not convict the previous one of what the " Times" calls " enormous lying ;" we are certain it will fail to " convert" any who were not previously converted from their hostility to the " better system" from which so much was anticipated, and from which so much that was beneficial did actually result, according to the Assistant Commissioners.

" We think it is incumbent upon us again to allude to the arrangements respecting medical relief, which has so often been the subject of animadversion and complaint in communications addressed to your Lordship by different members of the medical profession.

It has been so perseveringly maintained that the medical relief to the poor has been inadequately provided for, and that a course has been pursued by us, injurious to the medical profession, that we think it necessary to re-affirm the statement which we have made whenever we have had a fit opportunity for doing so ; that it has been our wish and intention to provide adequately for that important branch of pauper relief ; and that in so doing we have never sought to disturb or displace the medical practitioners in their respective districts. Whenever this has been done (and the introduction of other individuals from a distance has occurred in but very few instances) the guardians have been forced to adopt that course by the inadmissible demands which have been made upon them by the gentlemen who now complain of their practice having been interfered with.

We wish indeed that it were possible for us, within the limits of this report, to exhibit to your Lordship a full and complete representation of all that has been done as to this branch of our duties ; and we think we should be able clearly to demonstrate that the complaints of the medical profession have no foundation.

No sooner, however, was any part of the country formed into Union, than the propriety of altering the arrangements established for the purposes of medical relief in the separate parishes, and of resorting to a new and more combined distribution, became apparent. The effecting of this object caused some disturbance to the medical practitioners, extending the practice of some, whilst it curtailed that of others. The division into districts has given rise to much complaint. The districts are represented by the medical practitioners as being generally too large, and as otherwise inconvenient. These districts, however, have been deliberately formed by the respective Boards of Guardians, who, from their local knowledge, must be considered to be the most competent judges on the subject; and we have reason to believe that in almost every instance the best arrangement was adopted.

Another ground of complaint is, that the medical gentlemen have, by the Guardians of some Unions, been called upon to make known, by the way of tender, the amount of payment for which they would undertake to supply medical attendance and the requisite medicines in any district which the Guardians might be desirous of providing for. Although this mode of proceeding is not rendered imperative by any thing contained in our rules, there is no doubt that the Guardians have generally resorted to it, and have by us, through our Assistant-Commissioners, been advised to do so; but it was never supposed that such a course was derogatory to the character of the profession. We are ready to admit that the principle of competition, though it is strictly, and to the fullest extent, applicable to the supply of drugs and many other articles used by the medical practitioners, is yet capable of only a modified application when brought to bear upon the acquired skill and knowledge, and other personal qualifications, the possession of which must and ought so materially to influence the Guardians in the selection they shall make from amongst the medical candidates; and in all our operations this limitation of the principle of competition has been recognized and acted upon; the Guardians having never been required to accept the lowest tender, and having in fact, in very many instances, been induced to set it aside solely with reference to the considerations of character and personal qualifications, to which we have alluded."

This was by way of apology for the treatment of the poor and the profession. It failed of its effect, the murmur of discontent grew louder, and Parliament was forced, by the petitions that crowded its table, to an inquiry.

This was instituted—a select Committee of the House of Commons was appointed—they sat, they took evidence—they made a report; and "highly respectable medical men" were no longer to drive a disgraceful bargain with paupers to save themselves from the dire necessity of subscribing to a more disgraceful contract with Boards of Guardians. Instead of half-a-crown a case being deemed adequate and ample remuneration, 6s. 6d. was not considered too large a sum to recommend as an equivalent for medical relief to any and every individual pauper in time of sickness.

The "*better system*" with all the Commissioners' sanguine "anticipations," and all the Assistant Commissioners' florid statements of benefits *said* to be realized, received its quietus from the Report of the select Committee of the House of Commons. We proceed to examine the EVIDENCE OF THE MEDICAL WITNESSES, upon which that Report is founded.

The witnesses examined were Sir Astley Cooper, Bart. Drs. Kay, an Assistant Poor Law Commissioner, Marshall Hall, Elliotson, Thomson, and Webster, and Messrs. Rumsey, Ceely, Rowe, Farr, Evans, and Toogood.

1, Sir Astley Cooper:—had known great evils arise from the districts being too extensive. One such district is nine miles long by four wide—a poor woman has her child taken suddenly ill with acute disease—she is obliged to go to the distance of nine miles to a medical man—he may not be at home—he may be in the opposite direction—it is a long time before his advice can be procured, and a still longer time before the medicine can be got. To remedy this double evil, he recommends the limitation of rural districts to a diameter of five miles, and the presence of a depôt for medicines in every parish.

Thinks the system of letting the medical offices " by tender," MOST HORRIBLE—that it is quite an absurdity for lay Guardians to attempt to form an opinion upon a medical case—that, it would certainly be satisfactory to the profession and useful to the poor, if there were some medical guardian appointed, who should be referred to in matters of that kind—and that 7s. per case would be about a fair remuneration for the medical attendant's labour, &c. in rural districts.

The Baronet's answers to two of Mr. Wakley's questions do him infinite credit, and the first of those answers we would have inscribed in letters of gold, and hung up in the Board Room of every Union throughout the kingdom.

" 16048. Do you consider it of importance that the medical practitioner in attendance upon the poor should be a man of some standing and residence in the district, and that he should also be in attendance upon the rich ?—*You ought to look out for the same man to attend upon the poor as you would be satisfied with in your own family*; MAKE THE CASE YOUR OWN AND YOU ARE SURE TO BE CORRECT."

" 16049. You do not think it advisable to introduce strangers to practise among the poor if there are resident practitioners who are willing to take the offices ? If a very clever man comes into that part of the country very highly recommended, I see no reason why you should exclude that medical man, even supposing there were resident practitioners there ; but if a medical man comes into the district and says, 'I will attend for £50 where the resident practitioner has offered to attend for £100,' *there can be nothing more horrible, or more degrading to the profession, or more injurious to the poor.*"

Sir Astley also considers three things " absolutely essential," as qualifications for office as parochial medical attendants upon the poor. 1st. The having passed the ordeal of the Apothecaries' Hall. 2nd. The possession of the diploma of the Royal College of Surgeons. And 3rdly. The having undergone an examination before a midwifery board, (no such board being now, nor ever having been in existence in this country.)

2. DR. MARSHALL HALL;—considers that medical attendance upon the sick poor involves a severe moral and professional responsibility;—that "offers by tenders" are derogatory to the medical profession; that much practical experience is necessary for the performance of the duty; that it should only be entrusted to men of two years' standing in the profession, acquainted with the particular locality, in the habit of attending upon the rich as well as the poor, resident in the centre of a district, and that district from 12 to 16 square miles; that, in electing officers, fixing the remuneration and prescribing the duties to be performed, a professional authority should be available for reference; that the medical attendant upon the poor amidst an ordinary population of 10 or 12,000 would have sufficient work to perform; that the poor, as a body, need, in acute diseases, even more attention than the rich; are more liable to contagious diseases; that it would be desirable to render the medical appointments more conducive to the advancement of science, more satisfactory to medical men, and more beneficial to the poor, than they are rendered at present; and that "*the remuneration should be such as no man should be ashamed of accepting, and such as no set of men should be ashamed of proposing.*"

We print this last opinion of the worthy Doctor in italics, for the especial benefit of all whom it may concern, including Poor-Law Commissioners and Assistant Poor-Law Commissioners, Peers of Parliament, and Members of the House of Commons, Boards of Guardians in particular, and rate-payers generally.

3. DR. JOHN ELLIOTSON:—thinks, the medical districts should not exceed 12 square miles, it being an act of justice to the poor that the medical districts should be as small as possible; that one reference should be medical with regard to all medical appointments throughout the kingdom; that the surgeons in rural districts ought not to have less than six shillings and six pence, or 7s. a case; that the system of "tender" is "most injurious and most improper in every respect," making medical men do in that particular what they condemn each other for doing in private practice, and being a temptation to men to attend to the poor far less than they otherwise would; that, no one ought to be allowed, however long he may have been in practice, to undertake the care of an extensive district without medical control.

The good sense and honesty of Dr. Elliotson are conspicuous in his answers to the questions 15869, 15870 and 15871; and we quote them entire:—

"15869. Would you recommend that a man should be of some standing in his profession, say two or three years, before he is permitted to take a situation the duties of which are of such a responsible character as those connected with the Poor Law Unions?—I doubt whether he would be more fit in 2 years, if he had not had a good opportunity of improving himself by practice, than at the beginning; because he would have so little private practice in the first two years, that he would not much improve himself by it; and at the same time he might have forgotten a good deal that he knew when he came from his examinations; a man must learn his profession by experience, as well as by education at the schools.

15870. If he had been in practice and had acquired additional experience in that way, would he not be more fitted to undertake the medical duties of a Union?—He would; but he would not perhaps be so able to devote himself to it as a young man who had no other patients.

15871. But still, he being of some standing, and resident in the neighbourhood, it is likely that he would be better acquainted with the circumstances of the poor, and that his own character would be better known to the inhabitants?—Certainly; and it would be more satisfactory to the poor than to see a person appointed whom they had never heard of before and who was perfectly a novice." —*Minutes of Evidence, &c.* p. 118.

The further examination of this physician elicited a remarkable testimony to the efficiency of the medical attendance upon the sick poor in London, so far as it has fallen under his own observation. He considers them to have been very well attended generally, and in the Hanover Square district, where he resides himself, believes them to have been attended in the most admirable manner.

His examination closes with the following sentence upon the abominable tender system, the "better system" of the Poor Law Commissioners, from which *they anticipated*, and their Assistants *found* so much good to result:—"I have spoken against it all my life, and those who have known others accept of it have shaken their heads with disgust; and again, judging from human nature in general, it must be impossible, I think, for a medical man to do his duty when he has an inefficient remuneration; it must be injurious to the poor; and it is heart-breaking to a man in any profession to work for nothing." p. 119.

4. Dr. Robert Dundas Thomson.—This gentleman's testimony went principally to satisfy the House of the vast extent to which drugs are adulterated for the purpose of meeting the prices offered by different sets of purchasers; and the inference from the facts adduced by him is, that the temptation to supply the poor with adulterated and worthless drugs, will increase in the ratio of the deficiency in any such provision made for their supply.

5. Dr. George Webster's evidence is only noticeable for an instance of impudent dictation and tyrannical exercise of authority, on the part of the Camberwell Guardians; and of cool and cavalier insolence on the part of the Poor Law Commissioners, for which we have no room, and must refer our readers to the "Minutes" themselves, while we pass on to—

6. Dr. James Phillips Kay, an Assistant Poor Law Commissioner, whose "testimony" we at first thought of consigning "to the tomb of all the Capulets," but on reconsideration have resolved to give him the benefit of a little wider notoriety than he at present enjoys. He slips away like an eel from every attempt on the part of his examiners to elicit truths from him, whether of fact or opinion, the full scope and tendency of which he may not happen to perceive, or perceives to be fatal to himself and the cause of which he is the paid champion. One example of this *slipperiness* will suffice to exhibit that quality of his testimony:—he is asked—

"5170. If a Union had engaged a medical man for six months, and had great reason to be satisfied with his conduct, you would not wish them to change him?—My only object is not to encounter those feelings of rivalry on the part of the medical practitioners, which interfere with their kindly relations in society." 14.

This answer does not appear to have contented the Member for Finsbury, and he tries his hand at skinning the eel.

"5171. (Mr. Wakley.) Do you not find that, when a medical officer anxiously discharges his duty, he in a short time obtains the confidence of the paupers; and do you not think it would be an evil to remove such a man from them?—I think the qualities of medical men resident in the Union are well known to the inhabitants, and if there were any person whose attendance was objected to by the poor, he certainly ought not to be a person selected for the duties of the workhouse."

Having glided thus dexterously from between the fingers of Mr. Wakley, the Chairman appears to have given him over as a bad job, and allows him to escape with the questions unanswered, by calling upon him for another Report which very properly winds up his examination—a Report, viz. of Mortality in the Workhouses. And even that he mystifies by making four quarterly returns instead of one annual.

The Doctor's memory is at times singularly leaky:—

"5083. Will you state the highest and lowest population in the two counties? (of Norfolk and Suffolk)—In casting my eye over the Suffolk medical districts, I do not find that any rural medical district contains a population greater than 6,800, which occurs in only one instance; I find that one town district, Ipswich, containing a population of 6,900.

5084. What is the salary of the medical officer in that district?—I cannot state that.

5085. What is the cost per head in that district?—I cannot tell as respects the particular medical officers' district; I could give information of the salaries respecting an entire Union."—*Minutes of Evidence*, p. 4.

Some important admissions, however, are wrung from the Assistant Commissioner, the first of which is, "I am not prepared to justify the present arrangements, excepting as provisional, and I expect changes to be made in the arrangements!" And a second, that:—"The time is past, for the operations of the system by tender."

And some very valuable facts are elicited, which if taken due advantage of, may lead to a more equitable adjustment of the balance between medical labour and medical remuneration—and to a juster settlement of all the medical questions involved in that one great subject—Poverty, *its* evils, and *their* remedies. Before concluding this article we shall notice summarily one of those evils—sickness; with a view to bring forward a remedy—adequate medical relief—and shall then recur to Dr. Kay's evidence.

7. EDWARD EVANS, Esq.—This gentleman's testimony relates to his own locality alone, (St. George the Martyr, Southwark,) and makes out a clear case of over-work and under pay—we say a clear case, and might add, a *common* one. Where, under the system, can a case be discovered of pay and labour equalized? He attends one half of a parish, the population of which is 40,000, and receives £110 per annum. The cost of drugs for his paupers averages £50 a year. The average number of *persons* attended by him within that period amounts to 1,229; and of *cases*, 2,059. The average remuneration for each case is—hear it, humanity! believe it, charity! make merry over it, political economy! embroider it on your pockets, every rate-payer in the kingdom! rejoice and be glad, Commissioners and Assistant-Commissioners! elected and ex-officio Guardians;—15729. *What is the amount of remuneration in each case?*—A SHILLING AND A HALF-PENNY IN EACH CASE, ATTENDING FROM SEVEN TO TEN DAYS."

Mr. Evans is asked, "15740. Have you much private practice?—I have considerable. I have been 22 years in the same neighbourhood. 15740. Have you much time to spare for private practice? My time is a great deal occupied by my private practice, as well as the parish practice."

We think the Guardians have exercised a very wise discretion in dividing the medico-parochial labours of St. George the Martyr, between four instead of two medical men. No two can attend to a "very considerable" private practice, without awfully neglecting so very considerable a parish practice.

But, of course, neither Guardians nor Commissioners think of a more equitable adjustment of remuneration to labour among the four than between the two. Increase the labourers! Yes, they are too few to do the work. Increase the amount to be divided among them? No, they will still receive *one shilling and a half-penny per case*, for medicines and from 7 to 10 visits. What could they look for more—and what would become of the rate-payers' pockets, if you gave them more?

8. George Robert Rowe, Esq.—This gentleman's evidence, like Mr. Evans', also makes out a strong case of ignorance, incapacity, and meanness, on the part of the officials connected in the medical arrangements of the Epping district. Ignorance, when not wilful, is alike pardonable and pitiable; to be angry with incapacity were to quarrel with creation, for "all men are not wise;" but meanness is a low vice which deserves to be whipped out of men and things whenever and wherever it is detected.

The surgeons of that district made an estimate, and offered to attend the poor at the rate of 2s. a head. They were met with a refusal, and a scheme offered to them of 2s. 6d. per head, per annum, for a man; 1s. 6d, for a woman; and 6d. for a child. This, with one exception, was refused by the rest of the medical men.

"15606. Was that for each case they attended, or for each individual of the pauper population?—Each individual in the receipt of weekly relief; but the Guardians reserved to themselves the privilege of increasing that number. The best way to illustrate it would be to give the population of my own parish; the pauper population of my own parish amounts to 440, or something more than 400; and in the paupers' schedule they comprised 100, that is 100 were the only persons receiving weekly relief; therefore, if any of the other 300 became sick, they had reserved to themselves the privilege of putting the sick patients into the schedule upon the same terms.

15607. (Mr. Wakley.) At 2s. 6d., 1s. 6d. and 6d. a head?—Yes, so that in the event of a family of six children, and a man and his wife, which is frequently the case in our district, being attacked by sickness, the medical man was called upon to attend the poor family for 7s.

15608. For the whole year?—Yes. 15609. (Chairman.) Whom do you mean by paupers?—Those who are not capable of maintaining themselves when they are sick. 15610. Do you mean to describe a man as a pauper who is not in the receipt of parish relief?—I consider that the moment the man applies to the parochial officers for medical relief, the man becomes a pauper. 15611. No doubt if the man applies for relief of any description and receives it, he becomes a pauper; but were those 400 in the receipt of relief of any description?—Only 100 were in the receipt of medical relief, but the other 300 were entitled to call upon the medical man for assistance, because their necessities were of that nature, that they could not provide it themselves. 15612. How do you know that?—From my own connexion with them; they were always considered paupers by me, from my having attended them for so many years. 15613. Then the state of things is this; 100 persons were receiving parochial relief, and, in your estimation, 300 other persons were in such circumstances that if sickness attacked them they would be under the necessity of applying for medical relief? Yes, I am quite sure of that. 15614. But the number of paupers, strictly speaking, were limited to 100?—Yes. 15615. And the remaining 300 were persons in such circumstances as to render it probable that, in the event of their becoming ill, they would not be able to provide medical relief for themselves?—Not only probable, but certain. 15616. Who formed an opinion upon that subject?—This was a scheme suggested for our approval by the Board, under the direction of the Assistant Commissioner, Mr. Power. 15617. Did the Board of Guardians come to this decision: that whereas, 100 persons were regular paupers in the receipt of constant parish relief of some description or another, 300 other persons were in such circumstances as to entitle them to medical relief in the event of their being ill?—No; they considered that those 300 were liable to become paupers the moment they were sick, because they made the provision for it; in fact, *they made the medical man liable to attend* 400 *paupers, and paid for only* 100*!!*"

It will be admitted we presume, that at such a rate the Doctors came off badly enough—and we presume there will be as little difficulty in admitting that the pockets of the rate-payers were tolerably well protected by Guardians and Assistant-Commissioners between them. But how fare the poor under this improved economical administration of medical relief?

The medical men of the district refused the terms of the Guardians and the Assistant Commissioners. What happened? Two out of the "plenty of talented young men from Somerset House," were sent down to supplant the resident practitioners.

"15667. What became of the officers who were in the Ongar and Epping Unions, and who had displaced the resident medical practitioners?—There were two of them; one was dismissed in consequence of a Coroner's inquest in the Epping Union; and another was dismissed in consequence of another Coroner's inquest! 15668. (Chairman.) What was the nature of those inquests?—They were inquests on the bodies of persons that they had attended; I do not know the particulars!! 15669. Do you happen to know whether there was anything in the inquests reflecting upon their treatment of the cases?—I presume there was, or they would not have been dismissed!!!"

Need we repeat our question, how do the poor fare under the "better system?"

We pass on to the evidence of

9. JONATHAN TOOGOOD, Esq. of Bridgewater.—According to this gentleman, there is one medical district in his neighbourhood, seventeen miles in extent, containing fourteen parishes, and part of it ten miles from the medical officer—besides this fact, there is nothing material elicited by his examination. His opinions coincide with those of other witnesses respecting the extent at which a rural district should be fixed, and the remuneration per case to be awarded to the medical officers.

10. ROBERT CEELY, Esq. of Aylesbury.—Admits that the "tender" system is no novelty, and that, in his neighbourhood, where it was in use before the Poor Law Amendment Act came into operation, "the medical men were more to blame than the authorities." He furnishes a laughable exhibition of the ignorance of Mr. Assistant Commissioner Gilbert:—

"15293. [*Chairman.*]—Do you know whether Mr. Gilbert had any personal communication with the medical men of the district?—He had not; *he fancied, in the first formation of the Union, that one medical man could do the duty of the whole Union;* he did not make that as an official communication, but he made it to a Guardian privately, who told him no such thing could take place; I had this from a person in communication with him.

"15294. [*Mr. Wakley.*] You have no doubt that he expressed that opinion?

—Not the slightest. 15295. What is the extreme length of the Union?—*The extreme length, in one direction, is twenty-three miles, and in the other direction, fifteen!*" p. 6

One of the expenses incidental to a medical appointment is also forcibly stated. On one occasion Mr. Ceely attended a parish in which an epidemic prevailed, and it cost him £10. to furnish wine and beef-tea for the patients because he was anxious to get them well as soon as he could; he was not compelled to do it; but the cases were so numerous and so distressing at the time, that he could not withhold that assistance.

Mr. C. thinks it desirable for a person who is practising amongst the higher classes to be aware of the epidemics that are prevailing among the poor; it is valuable to the poor, useful to himself, and advantageous to the community. He is also of opinion that greater facilities should be afforded to the poor for obtaining medical assistance than now exist; and sees, as an objection to payment per case, that *it restrains Guardians from giving orders for medical relief.*

Our space does not allow of our going at length into the examination of this witness—it will well repay any one who has inclination and leisure to peruse it in the "Minutes of Evidence" themselves. So far as we have yet passed those Minutes under review, he stands out conspicuous as one who makes the pockets of rate-payers, and even the reward of medical labour, very secondary considerations in comparison with the efficient medical relief of the sick poor. We have not the pleasure of a personal acquaintance with Mr. Ceely, but his evidence on this occasion has convinced us that he is what others would be thought to be—a lover of his kind, and a friend of the poor, in deed, as well as word.

11. William Farr, Esq.—This gentleman has been long and favorably known to the profession as a talented statistician; and by this time the whole country is in possession of no mean specimen of his ability in his letter to the Registrar General, published in that functionary's first report.

Through Mr. Wakley, all the returns from the different Unions were submitted to him—and the result of his examinations of them are given in evidence. According to that—the number of Unions is 501, the area of those Unions in square miles is 39,205, and the area of each Union in square miles is 78. 314 Unions contained 1,137 medical districts, consequently, each Union is divided, on an average, into 3·62 medical districts; that is 100 Unions are divided into 362 medical districts; 78 square miles, divided by 3·62, gives the average area of the medical districts, and from that it appears that the area is about $21\frac{1}{2}$ square miles.

It is obvious that a very considerable disproportion must subsist in the size of different Unions. Two of the larger are mentioned—Leighton Buzzard is 55, and Newbury 72 square miles. In both, the surgeons reside in the centre of their districts, removed eight miles in the one case, and seven and a half in the other, from the extremities.

"These numbers do not give the extreme points, but the distance North, or South, or East, or West; if, therefore, the greater distance should occur between these points, it would not be given. In the Wallingford Union the area of the medical district is 26 miles; the boundary of the Union is eight miles from the surgeon's residence in one direction. In the Workingham Union district No. 1, the area is 25 square miles; but the surgeon residing out of the district, the distance from his residence, in one direction, is eight and a half miles. In Devonshire, the districts are generally small, but the Oakhampton Union district No. 2, is 54 square miles in area, and the boundary is eight miles from the surgeon's residence in one direction. In Northleach, in Gloucestershire, the upper medical district is not distinguished from the lower, and the area of both is 109 square miles. The surgeon resides out of the district apparently; it is eleven miles from his residence, to the southern extremity. Districts 1 and 4 of the Kington Union, Herefordshire; Mr. Merrick was the surgeon in 1837. The area is not given in square miles, but the distance from the surgeon's residence in one direction is only one mile, in another ten miles; in the Ledbury Union, district 2, the surgeon appears to reside out of the district, the distance from his residence is eleven miles in one direction, ten in another; in the Leominster Union, district 3, the area is twenty-five square miles; but the surgeon residing out of the district, the distance of the boundary from his residence in one direction, is ten miles. There are few returns from Lancashire, I suppose but few Unions have been formed; but the distance from the surgeon's residence in the West Broughton district is eleven miles in one direction; and in the Cotton district, ten miles in one direction. In Norfolk, the Thetford Union, comprising parts of Thetford and Hopton, the distance from the surgeon's residence is eleven miles and a half in one direction." pp. 97, 100.

"In Northumberland, the Haltwhistle Union, East and West division, comprises an area of 108 square miles." "In Salop the distance of the boundary of district 2, Clun Union, in one direction is eleven miles, in another four, in another 5, in another 6; in Westmoreland, Shap Westward comprises 98 square miles; the distance from the surgeon's residence in one direction, nine miles; in York, the Driffield Union comprises 115 square miles; its distance from the surgeon's residence in one direction is nine miles; in another, six; in another, six; in another nine." p. 100.

The unnecessary expense of time and labour to the poor, and to the profession, by such an arrangement of districts is obvious. The peril to the sick poor, in every case of emergency, requiring prompt attention, must be equally obvious. Mr. Farr's statistical researches help us to a still further knowledge of the impossible duties assigned to the medical officer in populous as well as extensive districts. The Leighton Buzzard and Newbury Unions already referred to, contained in 1831—the former, 11824, the latter, 18,999 inhabitants. The number of cases treated in the former was 1,845, the number constantly sick and constantly under the care of the medical officer in Leighton Buzzard was 100; in Newbury Union, 93. If those persons were seen every other day, the one officer would have to visit daily 50 persons, scattered over an area of 55 square miles, and the other 46, similarly scattered over an area of 72 square miles. The Dover medical district comprises a population of 20,507; that of Sevenoaks and Shoreham, 13,735; of Leicester No. 1, 23,954; No. 2, 13,74. The Lincoln Union, district No. 1, comprised 65 parishes, and a population of 25,988; the number of cases was 550, constantly sick 20. The Bethnal Green Union, having three districts, had in 1831—62,018 inhabitants. In St. Martin in the Fields the population was 23,970—1,511 cases were treated; 125 were constantly on the sick list in 1837. The Lambeth Union, divided into 6 districts, with a population of 87,856; produced 16,450 cases in one year, and furnished 313 persons for the constant care of the medical officers.

The evil consequences of imposing such Herculean labours upon individuals who have besides to maintain themselves out of their practice, are as obvious as those resulting from the enormous extent of the rural districts. The sick poor, if not absolutely in a great number of instances, neglected, are subject to be mal-treated from the haste with which their cases must be disposed of; or the medical officer is driven to evade the performance of his too onerous duties by devolving their discharge upon an unqualified, or upon an ill-qualified assistant; or upon apprentices, for whom the parish affords fair practice. As for the poor, no body cares about them; and Sir Astley Cooper's triple remedy—the *ordeal* of the Hall *passed*, the *diploma* of the College *possessed*, and an *examination* before a Midwifery Board *undergone*, will fail to protect them from the boys and young men who dispense the medicines of the rich, and prescribe for the diseases of the poor in a vast multitude of instances. Frightful would be the result displayed, were the comparative mortality ascertained, in districts attended by the medical officer himself, and in those where he only exercises a general supervision over the attendance of his apprentices.

Let us proceed with the invaluable testimony of Mr. Farr; and ascertain the remuneration doled out to the labourers in fields so crowded and so extensive.

" The payment is generally per case, or by salary, or on the pauper schedule. The salaries vary exceedingly in different districts without any apparent cause." p. 105.

In Devonshire, for example, " in the Crediton Union, district 1, the payment per case is 3s.; in the 2d district of that Union, the payment per case is 1s. 9d.; if the number of cases exceed 88, 1s. 6d.; in the 3rd district it is 1s. 9d. per case also, and 1s. 6d. if the number of cases exceed 172; in the 4th district the payment per case appears to be 6s.; the difference in the area of the two districts does not account for that difference, for the area of district 3, is 16 square miles, and the area of district 4 is 21 square miles. In district 5, the payment per case is 5s., and if the number of cases exceeds 98,—4s. In district 6—6s., and above a certain number, 8s. In district 7—6s., and for all above 98—5s."

In districts 1, 5, and 8, 9 of the Kingsbridge Union, the payments are 10s. and 8s. with a fixed maximum however. In the Tiverton Union, workhouse cases are 3s. Town cases 2s. Parish cases (without the gates) 5s. Halberton 5s.; Uffculm 4s.; but if the number exceeds 160—3s.; Cullompton, 3s.; if the number exceeds 226—2s. 6d.—in the workhouse, 2s. 6d.; in Bradninch 3s., and 2s. 6d. if the number exceeds 134. In the Bampton, while under 270—5s.; in the Silverton 5s.; in the Thorverton 5s.; in the Washfield 6s.; in the Cleyhanger 5s. " The payment per case in Devonshire ranges from 1s. 6d. which is of course much too low, to 8s. and 10s. per case, but in nearly all those districts a maximum is fixed."

We are glad to coincide with Mr. Farr, that the payment per case system is radically bad; it is bad for the poor, it is bad for the practitioner.

The unwillingness of the relieving officers to grant orders under this system, and the calamitous consequences of such unwillingness are notorious. " In Devonshire, half the pauper population of the county appears to be attended on the per case system; in the other half of Devonshire the medical officers are paid by fixed salaries; the annual proportion of the cases to a population of 1000 in Unions where surgeons are paid by salary, is 68.

There are 68 casss out of a population of 1000 treated by the parochial surgeon; in the districts where the surgeons are paid per case the proportion is about 50, or rather under 50. *The mortality among the latter cases is much higher; the mortality among the cases attended per case is* 59 *per* 1000*; among the others only* 52 *per* 1000*;* 332 *deaths occurred in Devonshire among* 5587 *cases treated on the per case system; if the mortality had been the same as among the other cases, only* 292 *deaths would have occurred!* or 40 less than the actual number of deaths recorded."

So far the unwillingness of relieving officers to grant orders may be said to be demonstrated by Mr. Farr's figures. Had that gentleman possessed experience of the working of the system, even where the payment is by fixed salary, he would have been able to add that orders are multiplied to a medical officer in the inverse ratio of that functionary's attention to his duty and care of the poor.

If the medical officer leaves his pauper practice to apprentices and servants—the paupers die, it is true, in greater numbers; but then the parish is not burthened but rather relieved. If the medical officer sees his own sick, he finds they need wine and nutritious diet—he wishes their recovery—he recommends more liberal supplies of medical comforts than his more careless colleague—he loses fewer patients, but then the parish is more heavily taxed. Of what value is the poor man's health or life, weighed in the balance with the rich man's pockets.

Mr. Ceely need not have apologized for introducing the following quotation from Lord Bathurst—which is literally applicable to the case before us, and which, therefore, literally, and without apology, we apply to it in this place. Corporate bodies, have, indeed, no souls! "All corporations of men," observes Lord Bathurst, writing to Swift, "are perpetually doing injustice to individuals. I have often reflected (from what cause it arises I know not) that though the majority of a society are honest men, and would act separately with some humanity, and according to the rules of morality, yet, conjunctively, they are hard-hearted determined villains!"

We should be well content to transfer the whole of Mr. Farr's upright, manly, noble testimony to our own pages, and consent most grudgingly to let any of our readers go, with only part of it—but time and the printer both forbid.

According to Mr. Farr, and according to universal common sense, good medicines and good medical advice are more economical than bad medicines and bad advice. According to him, and according to common humanity, medical relief "cannot lead to the same abuse as relief in aid of wages, or as relief afforded when the labourer is out of work; no one will break his leg willingly, or take physic as an amusement; medical relief has no tendency to increase sickness or the demand for relief that cannot be refused. NO ONE WITH A HUMAN HEART CAN DENY RELIEF TO THE SICK: MEDICAL MEN CANNOT."—*Minutes of Evidence*, p. 107.

"No one with a human heart can deny relief to the sick; medical men cannot." That may be, but sad experience has proved that Commissioners and their Assistants, Boards of Guardians, and their Relieving Officers, CAN. As Lord Bathurst says, "CORPORATE BODIES HAVE NO SOULS."

It would, however, be unfair, not to add that Mr. Farr admits having had every facility given him in consulting the returns at Somerset House, and whenever he called upon Mr. Chadwick, the most ready access afforded him to those returns. Lux ex non ——; Spes ex non: let us look for the dawning of a better day, but let us also be up and doing, lest, if it be thought that we have again retired to our dormitories, while we sleep the plunder of the profession, and the mal-treatment of the poor, continue as before.

The last of the band of worthies who have helped to throw light upon the one evil of poverty to which the present article is devoted, and its remedies —namely—sickness—and medical relief, is:—

12. Henry Wyldbore Rumsey, Esq.—Who is also the author of one of the works which it was proposed to bring under review in this article. An analysis of his evidence will serve for a review of his pamphlet, which we recommend to every one who may be interested in the struggle between the rich and the poor, who help to pay all rates, without the name of paying any.

The details, with respect to extent of district—amount of labour exacted—and amount of remuneration assigned, correspond with those already given in our examination of the other parliamentary witnesses. He however, adds to those details, a very instructive table of the reduction in the number of medical officers in 12 Unions: "In the Aylesbury, from 16 to 3; in the Banbury from 14 to 3: in the Cookham Union, from 8 to 2; in one district of the Eton Union, from 4 to 1; in the Faversham, from several to 1; in the Newbury, from 12 to 1; in the Ongar, from 10 to 4; in the Shipston-on-Stour, from 10 to 2; in the Stow, from 8 to 3; in the Tonbridge, from 5 to 1; in one of the districts of the Windsor Union, from 4 to 1; in one district of the Witney Union, from 8 to 2; in the Woodbridge Union, from 10 to 4; *making a total of* 109 *reduced to* 27; *that is a deficiency of* 82 *medical officers out of* 109!"—*Minutes of Evidence*, pp. 28—9.

He also gives the lie to that "Report" which we have exposed already. Mr. Rumsey not only disputes the wisdom of the Commissioners' "better system," and denies their conclusions, he also contradicts their facts.

We have now closed our notice of the evidence taken before a Select Committee of the House of Commons. That evidence convinced the Committee—that the medical districts were too large—and the labours of the medical officers too onerous—that the system of contract by tender is not to be endured, and that the present average rate of remuneration, say 3s. 3d. per case, should be doubled.

Some of the recommendations of the witnesses, though not adopted by the Committee, are worthy to be reconsidered by any future Committee. These are—the deposit of a medicine chest, in every parish, where parishes are small and scattered, and many of them come to be included in a medical district—the appointment of a Medical Commissioner, and of Medical Assessors, for the better supervision and more effectual control of the medical department. Mr. Rumsey proposes that the election of the medical officer should be transferred from the Guardians to the practitioners at large, that, in fact, the medical officers should be elected as the Guardians themselves are elected.

We come now to consider Mr. Serjeant Talfourd's letter to Mr. Rumsey—and the proposed improvements which he is to bring before Parliament in the session 1839-40.

One of his propositions relates to the qualifications—another to the districts—and another to the remuneration of medical men. The two latter are easily settled, justice and mercy being the bases of the settlement. The former, he proposes, should consist of a College Diploma—and when the practitioner dispenses his own medicines, a Hall licence. This ought to conciliate Hall and College, and guarantees a fair qualification of ability.

The subject of medical relief for the sick poor has occupied our own thoughts for some years past. We have made extensive researches into the mode of administering it not only in this country, but also on the Continent, and have come to the conclusion that the present and all former modes among ourselves, is defective in principle, and vicious in practice.

This is not, however, the place for endeavouring to sketch a new, and a very different, system from that which has been hitherto adopted. At a future time, or in another place, we shall, probably, develope the views which we entertain, and which our crippled space prevents us from entering on at present.

In relation to medicine and the sick pauper, the Poor Law Amendment Act has hitherto proved a Pandora-box, from which evil after evil has come forth; who knows but that even from this fons malorum there may yet issue good at the last?

One word more. The poor are said by many to be sadly wanting in gratitude,—as though the poor were differently constituted from other men. Now we have not only always found with Boerhaave that the poor were our best patients because God was their paymaster—we have also found that the poor were more content with few attentions and a little kindness than the rich with much attendance and many good offices, and more grateful for ease and health than their wealthier neighbours. And we would need no better proof of a medical officer's having neglected or performed his duty, than his own appreciation of the spirit of gratitude evinced by his pauper patients. Uniting our experience with Mr. Ceeley, we adopt his language and say:—"We have found them very far from insensible to the advantages of a kind and close attention in cases of sickness—indeed the greatest satisfaction we have ever had in the performance of our duty has been with the poor, from the manner in which they received it, they appeared to appreciate it: nor, indeed, do we ever find so much gratitude evinced by any class of society as by the poor."

What a man sows that shall he reap. If the seed be charity, the wreath gathered will be gratitude.

